# Prognostic Impact of Visceral Pleural Invasion in Node-Negative Non-Small Cell Lung Cancer with Tumors ≤ 3 cm †

**DOI:** 10.3390/cancers18182944

**Published:** 2026-09-11

**Authors:** Ezgi Gürel Akan, Pınar Akın Kabalak, Suna Kavurgacı, Tuba İnal Cengiz, Funda Demirağ, Hakan Nomenoğlu, Hasan İbiş, Ülkü Yılmaz

**Affiliations:** 1 Department of Chest Diseases, Ankara Atatürk Sanatorium Training and Research Hospital, 06290 Ankara, Türkiye; 2 Department of Pathology, Ankara Atatürk Sanatorium Training and Research Hospital, 06290 Ankara, Türkiye; 3 Department of Thoracic Surgery, Ankara Atatürk Sanatorium Training and Research Hospital, 06290 Ankara, Türkiye

**Keywords:** non-small cell lung cancer, visceral pleural invasion, pathological staging, overall survival, progression-free survival, TNM classification

## Abstract

**Background and Objectives**: Visceral pleural invasion (VPI) up-classifies non-small cell lung cancer (NSCLC) tumors measuring ≤3 cm from T1 to T2; however, whether this stage migration translates into worse survival in pathologically node-negative disease remains uncertain. This study evaluated the prognostic impact of VPI on overall survival (OS) and progression-free survival (PFS) in this selected population. **Materials and Methods**: This retrospective single-center cohort study included patients who underwent surgical resection for pathologically node-negative NSCLC with tumors ≤ 3 cm between 2015 and 2021. In the primary analysis, patients with PL0 were compared with those with PL1–PL2, whereas PL3 cases were excluded from the primary comparison and evaluated only in exploratory analyses. Survival outcomes were assessed using Kaplan–Meier analysis and univariable and multivariable Cox regression models. **Results**: A total of 241 patients were included in the overall cohort (PL0, *n* = 179; PL1, *n* = 48; PL2, *n* = 9; PL3, *n* = 5). After exclusion of PL3 from the primary analysis, 236 patients remained, including 179 with PL0 and 57 with PL1–PL2. During follow-up, 58 deaths and 81 PFS events occurred in the primary analytic cohort. Kaplan–Meier analysis showed no significant differences between PL0 and PL1–PL2 in OS (log-rank *p* = 0.655) or PFS (log-rank *p* = 0.904). In multivariable analysis, VPI was not independently associated with OS (HR 0.785, 95% CI 0.389–1.582; *p* = 0.498) or PFS (HR 0.908, 95% CI 0.507–1.624; *p* = 0.744). Increasing age was independently associated with worse OS and PFS, whereas male sex and pneumonectomy were independently associated with worse OS. **Conclusions**: In surgically treated patients with pathologically node-negative NSCLC and tumors ≤ 3 cm, VPI defined as PL1–PL2 was not independently associated with OS or PFS. These findings suggest that the prognostic implications of VPI-driven up-classification may be context-dependent and should be interpreted together with other clinicopathological factors.

## Simple Summary

Visceral pleural invasion (VPI) can increase the T stage of small non-small cell lung cancers even when lymph nodes are negative. However, it is unclear whether this stage increase always reflects a worse prognosis. We evaluated surgically treated patients with tumors measuring 3 cm or less and pathologically node-negative disease. In the primary analysis, patients with PL0 were compared with those with PL1–PL2 pleural invasion. VPI was not independently associated with overall survival or progression-free survival. Exploratory analyses suggested that more extensive pleural involvement, particularly PL3, may have different prognostic implications, although the number of such cases was small. These findings suggest that VPI should be interpreted together with other clinicopathological factors rather than in isolation when estimating prognosis in patients with small, node-negative NSCLC.

## 1. Introduction

Accurate pathologic staging is essential for prognostic assessment and postoperative management in non-small cell lung cancer (NSCLC). In early-stage disease, even limited pathological findings may result in stage migration and influence treatment strategies. Among these factors, visceral pleural invasion (VPI) has particular clinical importance because it influences T classification within the TNM staging system and has been associated with adverse outcomes in several studies [1,2,3].

In the 8th edition of the TNM classification, visceral pleural invasion (PL1–PL2) upgrades tumors measuring 3 cm or less from T1 to T2. Consequently, in otherwise node-negative small tumors, VPI may become the sole factor responsible for stage upstaging. This is clinically important because such a change may alter postoperative risk stratification and influence consideration of adjuvant treatment. Notably, this principle has remained essentially unchanged in the 9th edition of the TNM classification, in which VPI remains a T2 descriptor [3,4,5,6,7].

However, whether VPI alone represents a uniformly meaningful adverse prognostic factor in small, node-negative NSCLC remains unclear. Although several studies and pooled analyses have associated VPI with worse survival in resected NSCLC, the magnitude and consistency of this effect appear to vary according to tumor size, nodal status, histology, and the extent of pleural involvement. In particular, studies focusing on small tumors have reported conflicting findings, with some suggesting a limited prognostic impact of VPI in highly selected early-stage disease, while others have shown an adverse effect on survival. Furthermore, increasing evidence suggests that pleural involvement should not be regarded as a biologically uniform entity. PL1 and PL2 constitute visceral pleural invasion, whereas PL3 represents extension beyond the visceral pleura and therefore reflects a distinct anatomical category. Even within VPI, the prognostic implications of PL1 and PL2 may not be equivalent [8,9,10,11].

This issue is particularly relevant in patients with tumors measuring 3 cm or less and pathologically node-negative disease, because in this subgroup VPI may serve as the sole determinant of T-stage upstaging. Clarifying whether this pathological finding is independently associated with inferior oncologic outcomes is therefore important for assessing the clinical significance of VPI-based upstaging in small tumors. Accordingly, the present study aimed to evaluate the association of visceral pleural invasion, defined as PL1–PL2, with overall survival and progression-free survival in surgically treated, pathologically node-negative NSCLC patients with tumors ≤ 3 cm. In addition, survival outcomes across the individual PL0, PL1, PL2, and PL3 categories were explored to assess whether prognosis differed according to the extent of pleural involvement [2,3].

## 2. Materials and Methods

### 2.1. Study Design and Patient Selection

This retrospective single-center study included patients who underwent surgical resection for non-small cell lung cancer (NSCLC) between 1 January 2015 and 31 December 2021. A total of 4075 patients who underwent lobectomy, pneumonectomy, or anatomical segmentectomy for NSCLC during the study period were identified from the institutional pathology records and individually screened for eligibility. After application of the predefined inclusion and exclusion criteria, 241 patients constituted the final study cohort. Patients were eligible if the primary tumor measured 3 cm or less and pathological nodal assessment showed node-negative disease. All tumors were retrospectively reclassified according to the ninth edition of the TNM classification for lung cancer. To ensure adequate outcome assessment, patients were required to have at least 2 years of follow-up unless recurrence or death occurred earlier.

Patients with missing pleural invasion assessment, incomplete clinicopathologic data, insufficient follow-up, or loss to follow-up were excluded. Patients who had received neoadjuvant treatment were also excluded. Molecular testing was available for all included patients, either at the time of surgery or subsequently during clinical follow-up, and patients with actionable driver alterations were excluded. Accordingly, none of the included patients received molecularly targeted therapy or immune checkpoint inhibitors during follow-up. To isolate the prognostic contribution of pleural involvement, patients with other non-pleural pathological descriptors independently resulting in T2 or higher classification were excluded.

Visceral pleural involvement was assessed histopathologically using elastic staining (Verhoeff–Van Gieson) when required for assessment of pleural invasion. Pleural invasion was classified as PL0, PL1, PL2, or PL3 according to established pathological criteria: PL0 indicated no invasion beyond the elastic layer; PL1 indicated invasion beyond the elastic layer without reaching the visceral pleural surface; PL2 indicated invasion to the visceral pleural surface; and PL3 indicated extension to the parietal pleura or chest wall. For the primary analysis, VPI was defined as PL1–PL2 and compared with PL0. Because PL3 represents extension beyond the visceral pleura and constitutes a distinct pathological category, PL3 cases were excluded from the primary binary analysis and retained only for exploratory analyses [12].

### 2.2. Ethics

This retrospective study was approved by the Scientific Research Evaluation Ethics Committee of Ankara Atatürk Sanatorium Training and Research Hospital, University of Health Sciences, Ankara, Türkiye (Ethics Committee Approval No: 65, Date: 29 January 2024). The requirement for informed consent was waived due to the retrospective nature of the study. All methods were carried out in accordance with the Declaration of Helsinki.

### 2.3. Data Collection

These included age, sex, histopathologic subtype, operation type, tumor location, tumor size, lymphovascular invasion, spread through air spaces (STAS), adjuvant chemotherapy, and details of pathological nodal assessment, including the numbers of examined N1 and N2 lymph nodes and nodal stations. At least three nodal stations were evaluated in each patient, and station 7 was sampled in all cases. Baseline clinicopathologic characteristics of the cohort are summarized in Table 1.

### 2.4. Study Endpoints

The primary endpoints were overall survival (OS) and progression-free survival (PFS). OS was defined as the time from surgery to death from any cause or the date of last follow-up. PFS was defined as the time from surgery to the first documented disease progression or death from any cause, whichever occurred first. Patients without progression or death were censored at the date of last follow-up. During follow-up, patients underwent routine thoracic computed tomography (CT). When recurrence or progression was suspected, further evaluation with PET/CT was performed, and histopathological confirmation by biopsy was obtained when clinically indicated and feasible. Recurrence/progression patterns were additionally categorized as local or systemic for exploratory analysis.

### 2.5. Statistical Analysis

Continuous variables were assessed for distributional characteristics using visual inspection of histograms and Q–Q plots. Non-normally distributed continuous variables were summarized as median and interquartile range (IQR) and compared between groups using the Mann–Whitney U test. Categorical variables were presented as numbers and percentages and compared using the chi-square test, Fisher’s exact test, or likelihood ratio test, as appropriate.

Overall survival (OS) and progression-free survival (PFS) were estimated using the Kaplan–Meier method and compared with the log-rank test. The primary Kaplan–Meier analyses compared PL0 with PL1–PL2, while analyses across the individual PL0, PL1, PL2, and PL3 categories were considered exploratory. Landmark survival probabilities at 2 and 3 years were reported with 95% confidence intervals. Median follow-up duration was estimated using the reverse Kaplan–Meier method. Numbers at risk were displayed beneath the Kaplan–Meier curves.

Univariable and multivariable Cox regression analyses were performed to evaluate factors associated with OS and PFS. Variables were first examined in univariable models. Given the limited number of outcome events, the multivariable models were deliberately restricted to a clinically justified set of covariates to reduce the risk of overfitting and improve model stability. Covariates were selected on the basis of clinical relevance, potential confounding, and univariable findings. The proportional hazards assumption was assessed using time-by-covariate interaction terms based on the logarithm of survival time.

Accordingly, age, sex, operation type, adjuvant chemotherapy, tumor location, tumor size, and visceral pleural invasion status (PL0 vs. PL1–PL2) were included in the final multivariable models. All selected covariates were entered simultaneously into the multivariable Cox regression models using the enter method. Female sex, lobectomy, no adjuvant chemotherapy, central tumor location, and PL0 were used as the reference categories. STAS and lymphovascular invasion were evaluated in univariable analyses but were not included in the final multivariable models to preserve model parsimony because of the limited number of outcome events and the absence of significant univariable associations.

Hazard ratios (HRs) with 95% confidence intervals (CIs) were reported. When evidence of non-proportional hazards was identified, an extended Cox model including the relevant time-dependent interaction term was examined as a sensitivity analysis. All statistical tests were two-sided, and *p* < 0.05 was considered statistically significant. All analyses were performed using IBM SPSS Statistics for Windows, version 24.0 (IBM Corp., Armonk, NY, USA).

## 3. Results

### 3.1. Baseline Clinicopathological Characteristics

Of 4075 surgically treated NSCLC patients screened for eligibility, 241 met the study criteria and constituted the overall study cohort. According to pleural involvement, 179 patients were classified as PL0, 48 as PL1, 9 as PL2, and 5 as PL3. For the revised primary analysis, PL3 cases were excluded, resulting in an analytic cohort of 236 patients, comprising 179 PL0 and 57 PL1–PL2 patients. During follow-up, 58 deaths occurred in this primary analytic cohort, including 46 in the PL0 group and 12 in the PL1–PL2 group. For progression-free survival, 81 events, defined as progression or death, were recorded, including 63 in the PL0 group and 18 in the PL1–PL2 group. Baseline clinicopathological characteristics according to the revised pleural invasion comparison are summarized in Table 1.

Median follow-up for the overall cohort, estimated using the reverse Kaplan–Meier method, was 64.0 months (95% CI, 59.5–68.5). In the revised PL0 versus PL1–PL2 comparison, median follow-up was 67.0 months (95% CI, 60.6–73.4) in the PL0 group and 62.0 months (95% CI, 56.6–67.4) in the PL1–PL2 group.

Baseline characteristics were generally comparable between the PL0 and PL1–PL2 groups, although several clinically relevant differences were observed. Median age did not differ significantly between groups (63 [IQR, 56–69] vs. 65 [IQR, 60–68.5] years, *p* = 0.378), and no significant differences were observed in sex, lymphovascular invasion, STAS, or the extent of pathological nodal evaluation. In contrast, histopathologic distribution differed significantly between groups (*p* = 0.002), with a higher proportion of adenocarcinoma and a lower proportion of squamous cell carcinoma in the PL1–PL2 group. Operation type also differed between groups (exact *p* = 0.032). Peripheral tumors were more frequent in the PL1–PL2 group than in the PL0 group (66.7% vs. 46.4%), whereas central tumors were more common in the PL0 group (53.6% vs. 33.3%) (*p* = 0.008). Tumor size was significantly greater in the PL1–PL2 group (median, 2.4 cm [IQR, 1.9–3.0]) than in the PL0 group (median, 2.0 cm [IQR, 1.5–2.5]; *p* = 0.020). Adjuvant chemotherapy was administered more frequently in the PL1–PL2 group than in the PL0 group (64.9% vs. 22.9%, *p* < 0.001). The median total number of examined lymph nodes was 12 (IQR, 11–14) in both groups (*p* = 0.916), with no significant between-group differences in the numbers of examined N1 nodes, N2 nodes, N1 nodal stations, or N2 nodal stations (all *p* > 0.05) (Table 1).

### 3.2. Overall Survival

**Figure 1:**
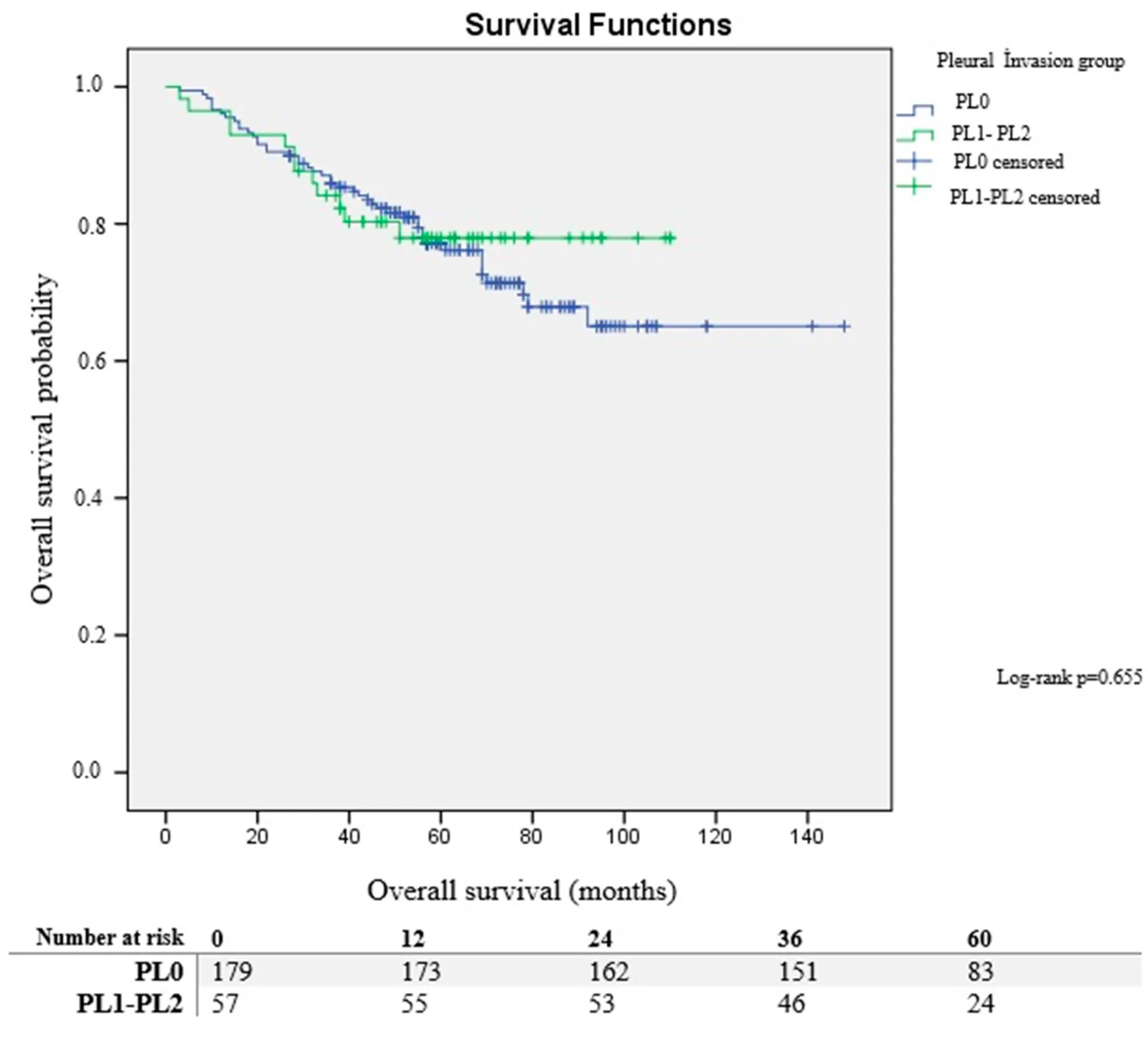
Kaplan–Meier estimates of overall survival according to PL0 versus PL1-PL2. No significant difference was observed between groups.

In the primary Kaplan–Meier analysis comparing PL0 and PL1–PL2, mean overall survival was 112.7 months (95% CI, 103.8–121.6) in the PL0 group and 91.7 months (95% CI, 82.4–101.0) in the PL1–PL2 group, with no statistically significant difference between groups (log-rank *p* = 0.655). Median overall survival was not reached in either group. The 2- and 3-year overall survival rates were 90.5% (95% CI, 85.1–94.0) and 85.9% (95% CI, 79.9–90.2), respectively, in the PL0 group and 93.0% (95% CI, 82.3–97.3) and 84.1% (95% CI, 71.5–91.4), respectively, in the PL1–PL2 group (Figure 1).

In univariable Cox regression analysis, older age (HR 1.061, 95% CI 1.025–1.097, *p* = 0.001) and male sex (HR 3.037, 95% CI 1.216–7.583, *p* = 0.017) were significantly associated with worse overall survival. Operation type showed borderline overall significance (*p* = 0.086). Compared with lobectomy, segmentectomy was not significantly associated with overall survival (HR 1.256, 95% CI 0.499–3.162, *p* = 0.629), whereas pneumonectomy was associated with worse overall survival (HR 3.677, 95% CI 1.145–11.808, *p* = 0.029). Adjuvant chemotherapy (HR 0.840, 95% CI 0.488–1.444, *p* = 0.527), tumor location (HR 0.820, 95% CI 0.492–1.365, *p* = 0.445), tumor size (HR 1.115, 95% CI 0.765–1.625, *p* = 0.571), STAS (HR 0.850, 95% CI 0.338–2.135, *p* = 0.730), lymphovascular invasion (HR 0.686, 95% CI 0.274–1.719, *p* = 0.421), and VPI status (PL1–PL2 vs. PL0; HR 0.866, 95% CI 0.458–1.634, *p* = 0.656) were not significantly associated with overall survival (Table 2).

In multivariable Cox regression analysis, increasing age remained independently associated with worse overall survival (HR 1.064, 95% CI 1.025–1.105; *p* = 0.001). Male sex was also independently associated with worse overall survival (HR 2.583, 95% CI 1.010–6.602; *p* = 0.048). Operation type was significant overall (*p* = 0.012). Compared with lobectomy, pneumonectomy was independently associated with worse overall survival (HR 7.315, 95% CI 1.949–27.447; *p* = 0.003), whereas segmentectomy was not (HR 0.772, 95% CI 0.262–2.274; *p* = 0.639). Tumor size (HR 1.237, 95% CI 0.813–1.884; *p* = 0.321), adjuvant chemotherapy (HR 0.812, 95% CI 0.443–1.489; *p* = 0.501), tumor location (HR 0.808, 95% CI 0.458–1.423; *p* = 0.459), and VPI status (PL1–PL2 vs. PL0; HR 0.785, 95% CI 0.389–1.582; *p* = 0.498) were not independently associated with overall survival (Table 3).

### 3.3. Progression-Free Survival

**Figure 2:**
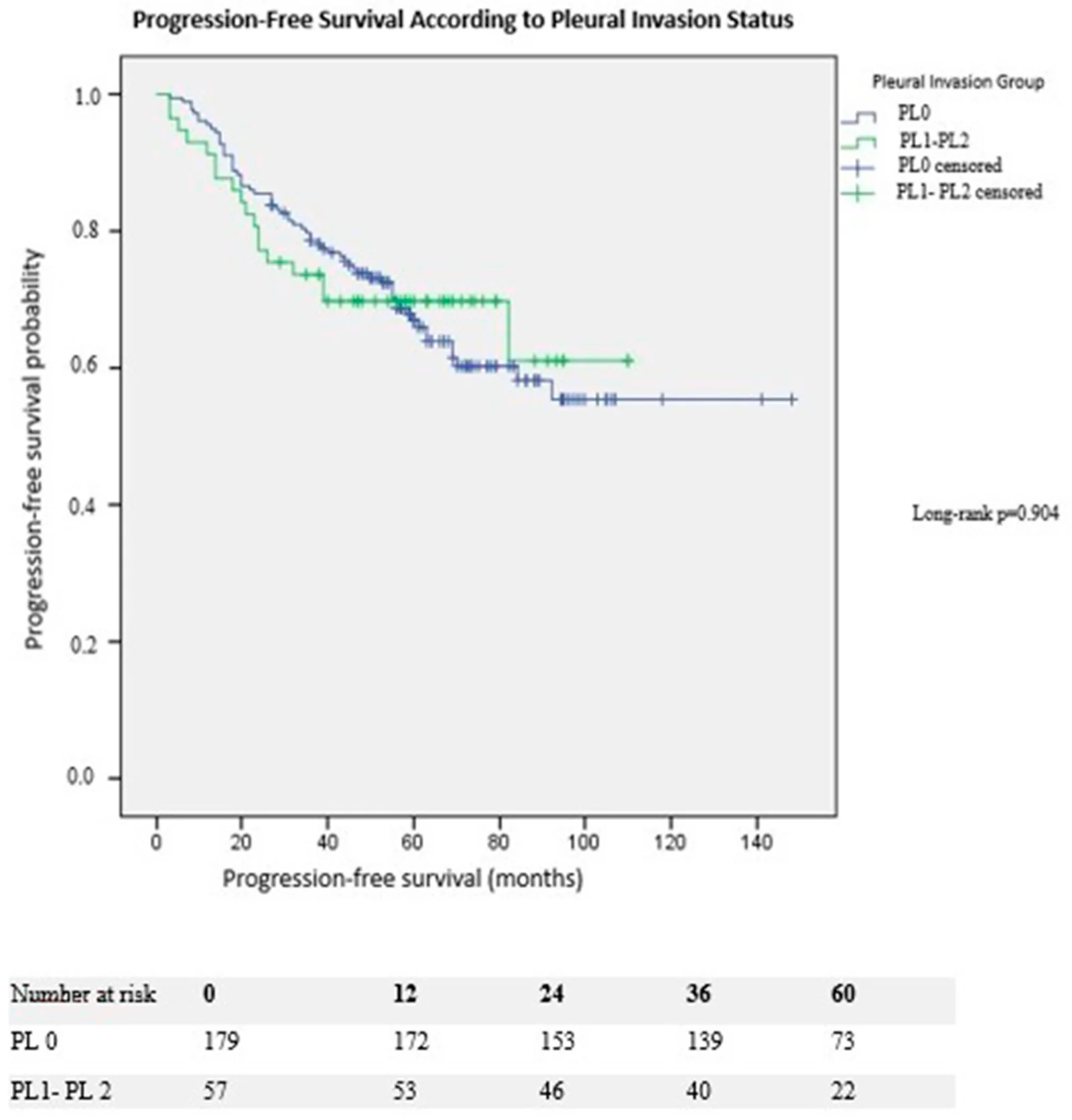
Kaplan–Meier estimates of progression-free survival according to PL0 versus PL1-PL2. No significant difference was observed between groups.

In the primary Kaplan–Meier analysis comparing PL0 and PL1–PL2, mean progression-free survival was 100.9 months (95% CI, 91.5–110.3) in the PL0 group and 80.1 months (95% CI, 68.7–91.6) in the PL1–PL2 group, with no statistically significant difference between groups (log-rank *p* = 0.904). Median progression-free survival was not reached in either group because fewer than 50% of patients experienced progression or death during follow-up. The 2- and 3-year progression-free survival rates were 85.5% (95% CI, 79.5–89.8) and 78.6% (95% CI, 71.8–84.0), respectively, in the PL0 group and 77.2% (95% CI, 63.9–86.1) and 73.6% (95% CI, 60.2–83.1), respectively, in the PL1–PL2 group (Figure 2).

In univariable Cox regression analysis, increasing age was significantly associated with worse progression-free survival (HR 1.038, 95% CI 1.010–1.067; *p* = 0.007), whereas male sex was not significantly associated with progression-free survival (HR 1.696, 95% CI 0.899–3.199; *p* = 0.103). Operation type was not significant overall (*p* = 0.335). Compared with lobectomy, neither segmentectomy (HR 1.102, 95% CI 0.477–2.546; *p* = 0.819) nor pneumonectomy (HR 2.380, 95% CI 0.749–7.565; *p* = 0.142) was significantly associated with progression-free survival. Tumor size (HR 1.129, 95% CI 0.821–1.551; *p* = 0.456), adjuvant chemotherapy (HR 0.931, 95% CI 0.591–1.465; *p* = 0.756), tumor location (HR 0.797, 95% CI 0.517–1.230; *p* = 0.305), STAS (HR 0.799, 95% CI 0.367–1.738; *p* = 0.571), lymphovascular invasion (HR 0.812, 95% CI 0.391–1.687; *p* = 0.576), and VPI status (PL1–PL2 vs. PL0; HR 0.968, 95% CI 0.573–1.635; *p* = 0.904) were not significantly associated with progression-free survival (Table 4).

In multivariable Cox regression analysis, increasing age remained independently associated with worse progression-free survival (HR 1.040, 95% CI 1.010–1.071; *p* = 0.009). Male sex was not independently associated with progression-free survival (HR 1.482, 95% CI 0.767–2.863; *p* = 0.241). Operation type was not significant overall (*p* = 0.106). Compared with lobectomy, segmentectomy was not significantly associated with progression-free survival (HR 0.718, 95% CI 0.276–1.872; *p* = 0.499), whereas pneumonectomy was associated with worse progression-free survival (HR 3.650, 95% CI 1.036–12.854; *p* = 0.044). Tumor size (HR 1.194, 95% CI 0.841–1.695; *p* = 0.321), adjuvant chemotherapy (HR 0.888, 95% CI 0.533–1.477; *p* = 0.646), tumor location (HR 0.763, 95% CI 0.475–1.223; *p* = 0.261), and VPI status (PL1–PL2 vs. PL0; HR 0.908, 95% CI 0.507–1.624; *p* = 0.744) were not independently associated with progression-free survival (Table 5).

### 3.4. Exploratory and Sensitivity Analyses

In exploratory analyses according to individual pleural invasion categories, overall survival differed significantly across the PL0, PL1, PL2, and PL3 groups (global log-rank χ^2^ = 9.271, *p* = 0.026), whereas progression-free survival did not differ significantly across the four groups (χ^2^ = 4.735, *p* = 0.192). For OS, the numbers of deaths were 46, 10, 2, and 3 in the PL0, PL1, PL2, and PL3 groups, respectively; the corresponding numbers of PFS events were 63, 15, 3, and 3. In pairwise OS comparisons, PL0 versus PL3 was significant (*p* = 0.003) and remained significant after Bonferroni correction for six comparisons (adjusted significance threshold, *p* < 0.0083). No other pairwise OS comparison and no pairwise PFS comparison remained statistically significant after correction. Given the small numbers of PL2 and PL3 cases, these subgroup findings were considered exploratory.

Because adjuvant chemotherapy use differed substantially according to pleural invasion status, exploratory survival analyses were additionally stratified by adjuvant treatment. Among patients who did not receive adjuvant chemotherapy (PL0, *n* = 138; PL1–PL2, *n* = 20), neither OS (log-rank *p* = 0.921) nor PFS (*p* = 0.668) differed significantly between PL0 and PL1–PL2. Similarly, among patients who received adjuvant chemotherapy (PL0, *n* = 41; PL1–PL2, *n* = 37), no significant differences were observed in OS (*p* = 0.812) or PFS (*p* = 0.785) between the two groups.

Recurrence/progression patterns were also examined according to pleural invasion status. Among patients with PL0, 22 (12.3%) developed local progression, and 13 (7.3%) developed systemic progression, compared with 6 (10.5%) and 5 (8.8%), respectively, in the PL1–PL2 group. The distribution of progression patterns did not differ significantly between groups (*p* = 0.886).

Assessment of the proportional hazards assumption showed evidence of non-proportionality for VPI status in the PFS model (VPI × ln[time] interaction, *p* = 0.024). Therefore, an extended Cox model including a time-dependent interaction term between VPI status and the logarithm of survival time was examined as a sensitivity analysis. The time-dependent interaction was significant (HR 0.452, 95% CI 0.226–0.902; *p* = 0.024), indicating attenuation of the effect of VPI on progression-free survival over time. Accordingly, the conventional Cox estimate for VPI should be interpreted with caution, although the analysis did not demonstrate a consistent adverse association between PL1–PL2 and progression-free survival.

Finally, an event-based sensitivity calculation using the Schoenfeld approach indicated that, given 58 OS events and 81 PFS events in the primary analytic cohort, approximately 80% power at a two-sided α level of 0.05 would correspond to detectable hazard ratios of approximately 2.36 for OS and 2.07 for PFS. These calculations indicate that the study was primarily capable of detecting relatively large effect sizes, whereas smaller clinically relevant associations may not have been detectable.

## 4. Discussion

In this study, we focused on a clinically selected subgroup of surgically treated NSCLC patients with tumors measuring 3 cm or less and pathologically node-negative disease, in whom visceral pleural invasion (VPI) could contribute to T-stage up-classification in the absence of other T2-defining features. The main finding was that VPI, defined in the primary analysis as PL1–PL2, was not independently associated with either overall survival or progression-free survival when compared with PL0. Consistent with the Kaplan–Meier analyses, VPI status was also not significantly associated with either endpoint in the adjusted Cox regression models. These findings do not challenge the established staging role of VPI; rather, they suggest that VPI-related T-stage up-classification may not necessarily correspond to a uniformly adverse prognosis in all small, node-negative tumors [13,14,15].

Previous studies have reported heterogeneous results regarding the prognostic significance of VPI in small, resected NSCLC, and accumulating evidence suggests that its clinical impact may depend on tumor size, coexisting pathological risk factors, and the extent of pleural involvement. Our findings are consistent with this heterogeneity, showing that VPI was not independently associated with worse survival among tumors measuring 3 cm or less with pathologically negative nodal status. However, this finding should be interpreted in the context of the limited number of outcome events. Event-based sensitivity calculations indicated that the study was primarily capable of detecting relatively large hazard ratios, whereas smaller clinically relevant effects may have remained undetected. Accordingly, the absence of a statistically significant association should not be interpreted as definitive evidence that VPI has no prognostic effect; rather, our results indicate that VPI did not demonstrate an independent prognostic association within this selected cohort [14,15,16,17,18].

In exploratory analyses according to individual pleural invasion categories, overall survival differed significantly across PL0, PL1, PL2, and PL3 groups, whereas progression-free survival did not. The significant OS difference was driven primarily by the PL0 versus PL3 comparison, which remained significant after Bonferroni correction. This finding should, however, be interpreted cautiously because only five patients were classified as PL3 and only nine as PL2, resulting in very limited statistical precision for the higher PL categories. Importantly, PL3 denotes extension beyond the visceral pleura to the parietal pleura or chest wall and is therefore pathologically distinct from PL1 and PL2; consequently, PL3 cases were excluded from the primary binary VPI analysis and retained only for exploratory category-specific analyses [18]. The low frequency of PL2 and PL3 was also consistent with the deliberately selected nature of the cohort, which was restricted to tumors measuring 3 cm or less with pathologically negative nodal status. Taken together, these findings suggest that the extent of pleural involvement may be biologically relevant, but the subgroup results should be considered hypothesis-generating rather than definitive [13,14,15,17]. 

Another notable finding was the independent association of increasing age with worse overall survival and progression-free survival. Male sex was also independently associated with worse overall survival, although it was not associated with progression-free survival. Operation type was significantly associated with overall survival in the multivariable model, with pneumonectomy showing a markedly higher risk of death compared with lobectomy. For progression-free survival, operation type was not significant overall; however, the pneumonectomy-versus-lobectomy comparison reached statistical significance. Given the small number of patients undergoing pneumonectomy and the wide confidence intervals around these estimates, these findings should be interpreted cautiously. Overall, these results emphasize that prognosis in small, resected NSCLC is influenced by multiple patient- and treatment-related factors and should not be inferred from pleural invasion status alone [19,20,21]. 

The implications of VPI for postoperative treatment remain controversial. Some studies have suggested that selected patients with stage IB disease and VPI may benefit from adjuvant chemotherapy, whereas others have not demonstrated a consistent benefit attributable to VPI alone. In our cohort, adjuvant chemotherapy was administered substantially more often in the PL1–PL2 group than in the PL0 group (64.9% vs. 22.9%), likely reflecting multidisciplinary treatment decisions in which pleural invasion was considered a high-risk pathological feature. Adjuvant chemotherapy was not independently associated with either overall survival or progression-free survival in the multivariable models. In addition, exploratory analyses stratified by adjuvant treatment showed no significant differences in OS or PFS between PL0 and PL1–PL2 among either patients who received adjuvant chemotherapy or those who did not [22,23,24,25,26,27,28,29,30]. Nevertheless, because treatment allocation was not randomized and was strongly associated with pleural invasion status, residual confounding by indication cannot be excluded.

Accordingly, our findings support a contextual rather than isolated interpretation of VPI in small, node-negative NSCLC. Pleural invasion should be considered together with tumor size, histopathological features, patient characteristics, surgical factors, and other established prognostic variables when estimating postoperative risk [29,31]. Although VPI remains an important component of T classification, our results suggest that its presence alone may not identify a uniformly high-risk subgroup within tumors measuring 3 cm or less. Given the retrospective design of the present study, these findings should not be interpreted as evidence for withholding or recommending adjuvant treatment on the basis of VPI status alone.

An additional consideration is that the proportional hazards assumption was not satisfied for VPI in the PFS model. In the extended Cox analysis, the significant interaction between VPI and log-transformed time indicated that the association between VPI and progression-free survival attenuated during follow-up. This finding suggests that the prognostic effect of pleural invasion on PFS, if present, may not remain constant over time. Therefore, the conventional Cox hazard ratio for VPI should be interpreted as a summary estimate rather than as evidence of a constant effect throughout follow-up. Importantly, the time-dependent analysis did not support a constant adverse association between PL1–PL2 and progression-free survival throughout follow-up, which is consistent with the overall interpretation of the primary analyses.

Another strength of the present cohort is the relatively consistent pathological nodal assessment. All included patients were pathologically node-negative, and at least three nodal stations were evaluated, with station 7 sampled in all cases. In addition, the total number of examined lymph nodes and the numbers of N1 and N2 nodes and nodal stations were comparable between the PL0 and PL1–PL2 groups. This reduces the likelihood that differences in survival were substantially influenced by an imbalance in the extent of nodal staging and supports the internal consistency of the primary comparison.

### Limitations

This study has several limitations. First, its retrospective, single-center design may have introduced selection bias and limits the generalizability of the findings. Second, although the cohort was deliberately restricted to tumors measuring 3 cm or less with pathologically node-negative disease to reduce clinical heterogeneity and isolate the prognostic relevance of pleural involvement, this focused selection limits extrapolation to larger tumors, node-positive disease, and more advanced-stage NSCLC. Third, the number of patients in the higher pleural invasion categories was small, particularly for PL2 and PL3, resulting in limited precision for category-specific analyses. Although overall survival differed across PL0, PL1, PL2, and PL3 groups and the PL0 versus PL3 comparison remained significant after Bonferroni correction, these findings should be considered exploratory because of the very small number of patients and events in the higher PL categories. Fourth, the relatively limited number of outcome events reduced the ability to detect modest prognostic effects. Event-based sensitivity calculations indicated that the study was primarily capable of detecting relatively large hazard ratios; therefore, smaller but clinically relevant associations cannot be excluded. Fifth, adjuvant chemotherapy was used substantially more frequently in the PL1–PL2 group, and although multivariable adjustment and treatment-stratified analyses were performed, residual confounding by indication cannot be fully excluded. Sixth, the proportional hazards assumption was not satisfied for VPI in the PFS model. Although an extended Cox model with a time-dependent interaction was used as a sensitivity analysis, this finding indicates that the effect of VPI on PFS may vary over time and that the conventional Cox estimate should be interpreted cautiously. Seventh, radiological variables such as ground-glass opacity proportion and consolidation-to-tumor ratio were not consistently available and therefore could not be incorporated into the analysis. In addition, PD-L1 expression was not routinely assessed during the study period and could not be evaluated as a potential prognostic variable. Finally, despite the focused cohort design, detailed pathological nodal assessment, and multivariable adjustment, residual confounding cannot be completely excluded in a retrospective analysis.

## 5. Conclusions

In surgically treated patients with NSCLC tumors measuring 3 cm or less and pathologically node-negative disease, VPI defined as PL1–PL2 was not independently associated with overall survival or progression-free survival. Although VPI remains an established staging descriptor, its prognostic impact may be context-dependent within this selected population and should be interpreted alongside other clinicopathological factors. Larger, multicenter studies with greater numbers of higher PL categories and outcome events are needed to further clarify the prognostic significance of VPI in small, node-negative NSCLC.

## Figures and Tables

**Table 1:** Clinicopathological characteristics according to the pleural invasion status.

*Primary Analysis: PL0 Versus PL1–PL2*			
**Variable**	**PL0** **(*n* = 179)**	**PL1–PL2** **(*n* = 57)**	***p *** **Value**
Age, years, median (IQR)	63 (56–69)	65 (60–68.5)	0.378
**Sex, *n* (%)**			0.232
Female	38 (21.2)	8 (14.0)	
Male	141 (78.8)	49 (86.0)	
**Histopathology, *n* (%)**			0.002
Adenocarcinoma	106 (59.2)	39 (68.4)	
Squamous cell carcinoma	71 (39.7)	13 (22.8)	
Other	2 (1.1)	5 (8.8)	
**Operation type, *n* (%)**			0.032 ^a^
Lobectomy	164 (91.6)	55 (96.5)	
Pneumonectomy	13 (7.3)	0 (0.0)	
Segmentectomy	2 (1.1)	2 (3.5)	
**Tumor location, *n* (%)**			0.008
Central	96 (53.6)	19 (33.3)	
Peripheral	83 (46.4)	38 (66.7)	
**Tumor size, cm, median (IQR)**	2.0 (1.5–2.5)	2.4 (1.9–3.0)	0.020
Lymphovascular invasion, *n* (%)	24 (13.4)	6 (10.5)	0.570
STAS, *n* (%)	21 (11.7)	8 (14.0)	0.645
**Adjuvant chemotherapy, *n* (%)**	41 (22.9)	37 (64.9)	<0.001
Total examined lymph nodes, median (IQR)	12 (11–14)	12 (11–14)	0.916
N1 lymph nodes, median (IQR)	4 (3–5)	4 (3–5)	0.241
N2 lymph nodes, median (IQR)	8 (6–8)	8 (6.5–9)	0.063
N1 nodal stations, median (IQR)	1 (1–2)	1 (1–2)	0.810
N2 nodal stations, median (IQR)	2 (2–3)	2 (2–3)	0.521

Note: Values are presented as *n* (%) unless otherwise indicated. Continuous variables are presented as median (interquartile range [IQR]) and were compared using the Mann–Whitney U test. Categorical variables were compared using the Pearson chi-square test, as appropriate. ^a^ The Fisher–Freeman–Halton exact test was used for operation type because of small expected cell counts. STAS, spread through air spaces.

**Table 2:** Univariable Cox regression analysis for overall survival.

*Primary Analysis: PL0 Versus PL1–PL2*		
**Variable**	**HR (95% CI)**	***p *** **Value**
Age, per year	1.061 (1.025–1.097)	0.001
Male sex (vs. female)	3.037 (1.216–7.583)	0.017
Operation type, overall	—	0.086
Segmentectomy vs. lobectomy	1.256 (0.499–3.162)	0.629
Pneumonectomy vs. lobectomy	3.677 (1.145–11.808)	0.029
Tumor location, peripheral vs. central	0.820 (0.492–1.365)	0.445
Tumor size, per cm	1.115 (0.765–1.625)	0.571
Adjuvant chemotherapy, yes vs. no	0.840 (0.488–1.444)	0.527
STAS, present vs. absent	0.850 (0.338–2.135)	0.730
Lymphovascular invasion, present vs. absent	0.686 (0.274–1.719)	0.421
VPI, PL1–PL2 vs. PL0	0.866 (0.458–1.634)	0.656

Note: HR, hazard ratio; CI, confidence interval; STAS, spread through air spaces; VPI, visceral pleural invasion. Reference categories were female sex, lobectomy, central tumor location, no adjuvant chemotherapy, absence of STAS, absence of lymphovascular invasion, and PL0. Hazard ratios for age and tumor size represent the effect per one-year and one-centimeter increase, respectively. Operation type was assessed as a categorical variable; the overall *p*-value is shown together with category-specific comparisons.

**Table 3:** Multivariable Cox regression analysis for overall survival.

*Primary Analysis: PL0 Versus PL1–PL2*		
**Variable**	**HR (95% CI)**	***p *** **Value**
Age, per year	1.064 (1.025–1.105)	0.001
Male sex (vs. female)	2.583 (1.010–6.602)	0.048
Operation type, overall	—	0.012
Segmentectomy vs. lobectomy	0.772 (0.262–2.274)	0.639
Pneumonectomy vs. lobectomy	7.315 (1.949–27.447)	0.003
Tumor location, peripheral vs. central	0.808 (0.458–1.423)	0.459
Tumor size, per cm	1.237 (0.813–1.884)	0.321
Adjuvant chemotherapy, yes vs. no	0.812 (0.443–1.489)	0.501
VPI, PL1–PL2 vs. PL0	0.785 (0.389–1.582)	0.498

Note. HR, hazard ratio; CI, confidence interval; VPI, visceral pleural invasion. The multivariable model included age, sex, operation type, tumor location, tumor size, adjuvant chemotherapy, and VPI status. Reference categories were female sex, lobectomy, central tumor location, no adjuvant chemotherapy, and PL0. Hazard ratios for age and tumor size represent the effect per one-year and one-centimeter increase, respectively. Operation type was assessed as a categorical variable; the overall *p*-value is shown together with category-specific comparisons.

**Table 4:** Univariable Cox regression analysis for progression-free survival.

*Primary Analysis: PL0 Versus PL1–PL2*		
**Variable**	**HR (95% CI)**	***p *** **Value**
Age, per year	1.038 (1.010–1.067)	0.007
Male sex (vs. female)	1.696 (0.899–3.199)	0.103
Operation type, overall	—	0.335
Segmentectomy vs. lobectomy	1.102 (0.477–2.546)	0.819
Pneumonectomy vs. lobectomy	2.380 (0.749–7.565)	0.142
Tumor location, peripheral vs. central	0.797 (0.517–1.230)	0.305
Tumor size, per cm	1.129 (0.821–1.551)	0.456
Adjuvant chemotherapy, yes vs. no	0.931 (0.591–1.465)	0.756
STAS, present vs. absent	0.799 (0.367–1.738)	0.571
Lymphovascular invasion, present vs. absent	0.812 (0.391–1.687)	0.576
VPI, PL1–PL2 vs. PL0	0.968 (0.573–1.635)	0.904

Note. HR, hazard ratio; CI, confidence interval; STAS, spread through air spaces; VPI, visceral pleural invasion. Reference categories were female sex, lobectomy, central tumor location, no adjuvant chemotherapy, absence of STAS, absence of lymphovascular invasion, and PL0. Hazard ratios for age and tumor size represent the effect per one-year and one-centimeter increase, respectively. Operation type was assessed as a categorical variable; the overall *p*-value is shown together with category-specific comparisons.

**Table 5:** Multivariable Cox regression analysis for progression-free survival.

*Primary Analysis: PL0 Versus PL1–PL2*		
**Variable**	**HR (95% CI)**	***p *** **Value**
Age, per year	1.040 (1.010–1.071)	0.009
Male sex (vs. female)	1.482 (0.767–2.863)	0.241
Operation type, overall	—	0.106
Segmentectomy vs. lobectomy	0.718 (0.276–1.872)	0.499
Pneumonectomy vs. lobectomy	3.650 (1.036–12.854)	0.044
Tumor location, peripheral vs. central	0.763 (0.475–1.223)	0.261
Tumor size, per cm	1.194 (0.841–1.695)	0.321
Adjuvant chemotherapy, yes vs. no	0.888 (0.533–1.477)	0.646
VPI, PL1–PL2 vs. PL0	0.908 (0.507–1.624)	0.744

Note. HR, hazard ratio; CI, confidence interval; VPI, visceral pleural invasion. The multivariable model included age, sex, operation type, tumor location, tumor size, adjuvant chemotherapy, and VPI status. Reference categories were female sex, lobectomy, central tumor location, no adjuvant chemotherapy, and PL0. Hazard ratios for age and tumor size represent the effect per one-year and one-centimeter increase, respectively. Operation type was assessed as a categorical variable; the overall *p*-value is shown together with category-specific comparisons.

## Data Availability

The datasets generated and analyzed during the current study are not publicly available due to patient confidentiality and ethical restrictions. De-identified data may be available from the corresponding author upon reasonable request and subject to institutional and ethical approval.
